# A label-free quantitative shotgun proteomics analysis of rice grain development

**DOI:** 10.1186/1477-5956-9-61

**Published:** 2011-09-30

**Authors:** Joohyun Lee, Hee-Jong Koh

**Affiliations:** 1 Department of Applied Life science, KonKuk University, Seoul 143-701, Korea/Plant Genomics and Breeding Institute, Seoul National University, Seoul 151-742, Korea; 2 Department of Plant Science, Plant Genomics and Breeding Institute, and Research Institute of Agriculture and Life Sciences, Seoul National University, Seoul 151-742, Korea

**Keywords:** MudPIT, Rice, Spectral Counts, Shotgun proteomics

## Abstract

**Background:**

Although a great deal of rice proteomic research has been conducted, there are relatively few studies specifically addressing the rice grain proteome. The existing rice grain proteomic researches have focused on the identification of differentially expressed proteins or monitoring protein expression patterns during grain filling stages.

**Results:**

Proteins were extracted from rice grains 10, 20, and 30 days after flowering, as well as from fully mature grains. By merging all of the identified proteins in this study, we identified 4,172 non-redundant proteins with a wide range of molecular weights (from 5.2 kDa to 611 kDa) and *pI *values (from pH 2.9 to pH 12.6). A Genome Ontology category enrichment analysis for the 4,172 proteins revealed that 52 categories were enriched, including the carbohydrate metabolic process, transport, localization, lipid metabolic process, and secondary metabolic process. The relative abundances of the 1,784 reproducibly identified proteins were compared to detect 484 differentially expressed proteins during rice grain development. Clustering analysis and Genome Ontology category enrichment analysis revealed that proteins involved in the metabolic process were enriched through all stages of development, suggesting that proteome changes occurred even in the desiccation phase. Interestingly, enrichments of proteins involved in protein folding were detected in the desiccation phase and in fully mature grain.

**Conclusion:**

This is the first report conducting comprehensive identification of rice grain proteins. With a label free shotgun proteomic approach, we identified large number of rice grain proteins and compared the expression patterns of reproducibly identified proteins during rice grain development. Clustering analysis, Genome Ontology category enrichment analysis, and the analysis of composite expression profiles revealed dynamic changes of metabolisms during rice grain development. Interestingly, we detected that proteins involved in glycolysis, TCA-cycle, lipid metabolism, and proteolysis accumulated at higher levels in fully mature grain compared to grain developing stages, suggesting that the accumulation of these proteins during the desiccation stage may be associated with the preparation of proteins required in germination.

## Background

Rice is an important model plant because of its importance as a food crop, and because its genome is both known and relatively small in size. Rice is a major cereal crop for human consumption, and starch accumulation and physiochemical properties are important determinants of eating quality. Seed quality is also a critical biological concern. Genetic studies and transgenic analyses have revealed the mechanisms and genes involved in starch accumulation [1,2]. Recently, the nature of allelic diversity in starch biosynthesis, which is related to eating quality, was analyzed via a transgenic approach [3]. Monitoring mRNA expression patterns during seed development may elucidate the molecular mechanisms of seed development [4-6]. Xu et al. (2008) monitored proteome expression patterns during rice grain filling stages (from 6 days after flowering to 20 days after flowering). They reported a comprehensive rice proteome analysis to detect and identify 396 differentially expressed proteins. From expression analysis, they detected that the substantially up-regulated proteins were involved in starch synthesis and alcoholic fermentation, and down-regulated proteins were involved in central carbon metabolism and most of the other functional categories/subcategories such as cell growth/division, protein synthesis, proteolysis, and signal transduction. Their results suggest that a switch from the central carbon metabolism to alcoholic fermentation may be important for starch synthesis and accumulation in the developmental process [7].

With advances in mass spectrometry, multidimensional protein identification technology (MudPIT), a shotgun proteomic approach, was developed for large-scale, high-throughput protein identification [8]. The benefits of MudPIT were first introduced in the context of plant sciences for the construction of rice leaf, root, and seed reference maps that included the most comprehensive proteome exploration available [9]. MudPIT has also been applied to analyses of the common bean (*Phaseolus vulgaris*), a non-model plant [10]. Although the mass spectrometry of MudPIT tends to be qualitative rather than quantitative, various methods for quantification in MudPIT have recently been developed [11-13]. In comparative analyses of protein expression, spectral count (SC), which assesses the total number of assigned MS/MS spectra for peptides from a given protein, is considered a *label-free *quantification method. Even though the estimated expression ratio for low-abundance peptides is more accurate when using the radiolabel quantification methods [14], SC is linearly correlated with protein abundance over a dynamic range of two orders of magnitude, and provides estimates of relative protein levels between samples comparable to estimates derived by radiolabel quantification [12,15]. With proper normalization of SC, the relative concentrations of proteins can also be estimated [16].

After the comprehensive report of the rice grain proteome expression during grain filling stages [7], the rice grain proteome expression during entire developing stages, including grain filling, desiccation phase, and fully mature grain has not been studied yet. Here, we performed comparative shotgun proteomic analysis of rice grain development including grain filling and desiccation process. When constructing a proteome reference map for rice grain development, the approach of a shotgun proteomics analysis facilitates the detection of differentially expressed proteins during grain development and provides information regarding the relative concentrations of all identified proteins. We present construction of an in-depth proteome reference map, monitoring the expression patterns of the identified proteins, and to detect proteins that are expressed differentially during grain development.

## Results and discussion

### Morphological changes of rice grains during development

**Figure 1:**
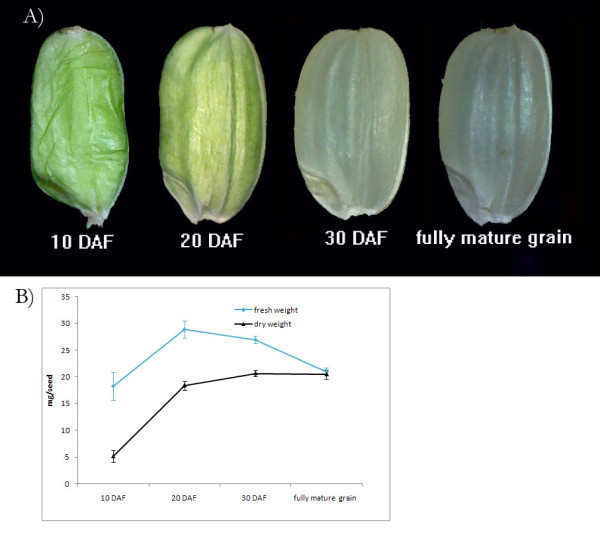
**Morphological changes of a rice grain during development**. A) Changes of the grain shape during grain development. B) Changes of the dry weigh and fresh weight of a grain during grain development.

The morphological changes of Ilpumbyeo rice grains are presented in Figure 1A). At 10 days after flowering (DAF), the grains almost reached their maximum length, but the grains were wrinkled. At 20 DAF, the overall shape of the grains was similar to that of mature grains, but they were green in color. No differences could be detected in overall appearance between 30 DAF and fully mature grain.

Both the fresh and dry weight increased drastically until 20 DAF and the dry weight was maximum in 30 DAF whereas the fresh weight was maximum in 20 DAF (Figure 1B), implying that starch accumulation lasted until sometime between 20 DAF and 30 DAF. This result was similar with the previous report by Kim et al (2011), where dry weight of Ilpumbyeo rice grains cultivated in the Korean natural field condition increased until ~25 DAF [17]. Since grain development varies depending on the variety of rice studied and environmental condition, the developmental process documented for Ilpumbyeo in Korea is different from that described in a previous rice grain proteome study by Xu et al. (2008), in which their proteomic analysis focused on the grain filling stage until 20 DAF.

### Constructing a large-scale rice grain proteome reference data set

**Figure 2:**
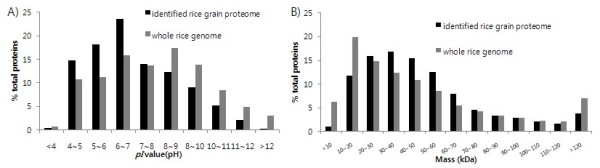
**Distribution of physiochemical properties of the identified rice grain proteome**. The the distributions of physiochemical proterties of the identified rice grain proteins were copared to those of total proteins encoded by the rice genome. A) Molecular weight. B) *pI *value.

We constructed a rice grain proteome database. Proteins were extracted from grains at 10 DAF, 20 DAF, 30 DAF, and fully mature grains. By merging all identified protein lists from all MudPIT runs including all four time points (10 DAF, 20 DAF, 30 DAF, and fully mature grain) and three biological replicates, we identified 4,172 non redundant proteins with a 0.05 false positive rate using PANORAMICS (refer to the material and method section)(Additional file 1). Among the 4,172 proteins, 889 proteins were identified in 10 DAF, 913 proteins were identified in 20 DAF, 1,095 proteins were identified in 30 DAF, and 899 proteins were detected in the fully mature grain. To increase the identification of hydrophobic transmembrane proteins, a mass spectrometry compatible detergent (dodecyl-β-maltoside) was used in the protein extraction buffer [10,18]. Using Phobius software (http://phobius.binf.ku.dk/), a total of 724 proteins (17%) among the 4,172 proteins were predicted to be transmembrane proteins. The distributions of molecular weights (MW) and *pI *values of the identified proteins were compared to those of proteins encoded by the rice genome (Figure 2). The MW of the identified proteins ranged from 5.2 kDa (LOC_Os04g02670.1) to 611 kDa (LOC_Os01g25450.1). The overall distribution of MW of the identified proteins was similar to that of the rice genome, even though low MW proteins were less prevalent. With regard to the *pI *values, the lowest pH was 2.9 (LOC_Os02g17860.1) and the highest pH was 12.6 (LOC_Os11g13934.1) for the identified proteins. Identified proteins with pH higher than 8 were less prevalent, and proteins less than pH 7 were more prevalent compared to those in the genomic distribution. However, the overall distribution of identified proteins was similar to that of proteins encoded by the genome, while 43% of the identified proteins were basic proteins (pH > 7), implying that the identification of the rice grain by MudPIT was not biased for the *pI *values.

To represent the overall trends of the specific functional categories that are enriched in rice grains, a Gene Ontology (GO) category enrichment analysis was conducted using all 4,172 identified proteins. The 4,172 proteins were categorized according to GO Slim classification for plants with the agriGO tool kit [19]. Among 4,172 proteins, 3,015 were annotated by this analysis and the GO of the remaining 1,157 (~28%) proteins were unknown. Among the annotated proteins, the GO category enrichment analysis revealed enrichments of 17 GO terms of biological processes (Table 1), 11 GO terms of molecular functions, and 24 GO terms of cellular components, that were significantly enriched in the constructed rice grain proteome (Additional file 4, Table S1). Since rice grains are reservoirs of carbohydrate, we detected significant enrichments of GO terms associated with the carbohydrate metabolic process, transport, and localization, suggesting that the constructed rice grain proteome represents the main cellular events of carbohydrate deposition during rice grain development. Furthermore, the enrichments of GO terms in the lipid metabolic process and secondary metabolic process suggested cellular event accumulation of lipids and secondary metabolites in rice grains. We detected the enrichment of GO terms related to cellular amino acids and the derivative metabolic process. In addition to the metabolic process, proteins involved in other biological processes were also enriched, such as translation, cellular homeostasis, signal transduction, and response to biotic stimuli, suggesting that cellular biochemical processes occurred during rice grain development. The enrichments of the GO terms of the catabolic process, and the generation of precursor metabolites and energy, are likely to be associated with the need for providing energy to these cellular biochemical processes. Interestingly, we detected the enrichment of the GO term associated with photosynthesis. The detection of proteins associated with photosynthesis was in accordance with the morphologies of the developing rice grains; the grains were green in color until 20 DAF. In general, the rice grain surface is not the main tissue of photosynthesis. Thus, one possible explanation may be that photosynthesis-associated proteins are over identified in plant proteomic experiments due to their abundance [20].

**Table 1:** Enriched GO terms of biological processes in the constructed rice grain proteome

GO term	Description	Number in the identified rice grain proteome	Number in rice genome	Adjusted *p*-value^*^
GO:0006519	cellular amino acid and derivative metabolic process	182	408	6.40E-55
GO:0005975	carbohydrate metabolic process	285	864	7.30E-52
GO:0009056	catabolic process	165	438	8.80E-39
GO:0006091	generation of precursor metabolites and energy	132	308	3.00E-38
GO:0006412	translation	214	683	5.10E-36
GO:0006810	transport	328	1639	4.10E-16
GO:0051234	establishment of localization	328	1639	4.10E-16
GO:0051179	localization	331	1658	4.10E-16
GO:0019725	cellular homeostasis	48	129	1.40E-11
GO:0042592	homeostatic process	48	136	1.10E-10
GO:0065008	regulation of biological quality	51	169	1.50E-08
GO:0006629	lipid metabolic process	103	492	1.30E-06
GO:0044267	cellular protein metabolic process	463	2983	1.30E-05
GO:0019748	secondary metabolic process	21	61	0.00011
GO:0015979	photosynthesis	27	100	0.00083
GO:0009607	response to biotic stimulus	8	18	0.0097
GO:0007165	signal transduction	50	268	0.024

*Based on the Fisher's statistical method and the Yekutieli FDR multiple test correction method [31]

Based on the annotation of Rice Genome Pseudomolecules Release V6.1, 12 proteins were annotated as hypothetical proteins. Under the hypothesis that these proteins play specific roles in grains, their mRNA expression patterns throughout the life cycle of the rice plant were searched using the Rice Expression Profile Database (RiceXPro: http://ricexpro.dna.affrc.go.jp/index.html). Only one gene, LOC_Os06g44190.1 reported expression that was higher during endosperm and embryo development, even though low level expression was detected in other tissues or organs. We suggest that this hypothetical protein may play a grain specific role in development. However, it is difficult to predict the roles of proteins using expression patterns, and therefore the specific role of this protein should be explored in future studies, such as knock out studies of the gene by the RNAi technique.

### Differentially expressed proteins during rice grain development

For the comparison analysis, the relative abundances of the identified proteins were obtained with the method of spectral count, a label-free method (refer to the material and method section). All 4,172 proteins were not reproducibly identified in all experiments including developmental stages and replicates due to analytical incompleteness in shotgun proteomic analysis, in which any single analytical run may only identify a fraction of the relevant peptides in a highly complex mixture of peptides [21]. Thus, for comparative analysis, we distinguished qualitatively expressed proteins from proteins that were not qualitatively expressed, but were identified only at certain time points due to analytical incompleteness. We included only proteins that were identified for all three biological replicates with at least two SCs for each replicate in the comparative analysis. After applying this criterion, 1,784 proteins were subjected to comparative analysis. The SCs for these 1,784 proteins were globally normalized (NSpC), followed by ANOVA test with logarithmically transformed NSpC (the natural log (Ln) of NSpC). The average coefficient of determination (*R*^2^) between NSpCs for the biological replicates was ~0.75, suggesting linear correlation. Among the statistically significant proteins detected by the ANOVA test, proteins with expression levels that changed less than two-fold were discarded. Following these strict criteria, we detected a total of 484 proteins that are differentially expressed during rice grain development (Additional file 2).

### Hierarchical clustering analysis

**Figure 3:**
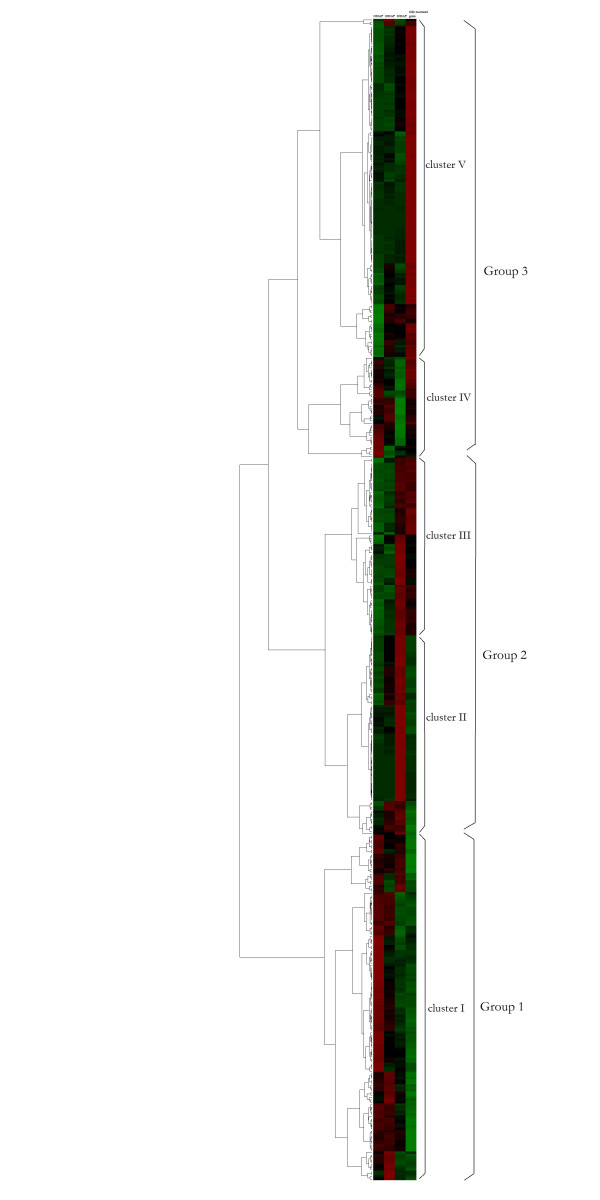
**Hierarchical clustering analysis for the 484 differentially expressed proteins**.

We conducted clustering analysis for the 484 differentially expressed proteins, and GO category enrichment analysis for proteins in selected clusters. Broadly, three main accumulation patterns were detected (Figure 3, Additional file 3). In the group 1 which includes the cluster I, most of proteins accumulated during the early development stage (until 20 DAF) and some of proteins were accumulated until 30 DAF, which represented the grain filling process. Twelve proteins associated with photosynthesis belonged to the cluster I. In the group 2 which includes the cluster II and cluster III, proteins accumulated in the end of the grain filling stage and the early desiccation phase. Proteins in the cluster II accumulated in 30 DAF and 5 of seed allergenic proteins were detected in this cluster. In cluster III, proteins accumulated in 30 DAF and fully mature grains, which represented the end of the grain filling stage and desiccation phase. Tree of late-embryogenesis-abundant (LEA) proteins were belonged to the cluster III. In the group 3 which includes the cluster IV and cluster V, proteins accumulated in fully mature grains, which represented the desiccation phase and proteins that may be required for germination. In cluster IV, proteins were accumulated in 10 DAF and fully mature grains. Interestingly, 25 proteins out of 42 proteins were associated with primary metabolic process. In cluster V proteins were accumulated in fully mature grains. Ten proteins associated with protein folding and 2 LEA proteins belonged to this cluster. The GO category enrichment analysis revealed that for the biological process, 31 GO categories, 43 GO categories, and 68 GO categories were significantly enriched in the group 1, group 2, and group 3 respectively (Additional file 5, Table S2). Enrichment of GO categories associated with photosynthesis was only detected in the group 1 which was in accordance with the appearance of early developmental grains. The enrichment of proteins involved in metabolic process was detected in the group 1, which represents the typical grain filling in the early developmental stage. Interestingly, enrichment of the GO category representing metabolic processes (GO:0008152) was also detected in other two groups, implying that even in the desiccating phase, changes occurred in molecular levels. In the group 2, several categories for stress responses, such as immune response and response to external stimulus, were enriched, which was possibly resulted from the stress condition in desiccation process. The GO category of protein folding was enriched in the group 2 and group 3, which may represent the important roles of chaperone proteins in rice grains. Since the role of rice grain is not only carbohydrate storage but also a seed (a resting body), the induction of chaperone proteins in late development stages (after morphological development of the embryo and endosperm) may be associated with conserving proteins associated with germination from desiccation stress.

### Composite expression profile of functional categories during rice grain development

**Figure 4:**
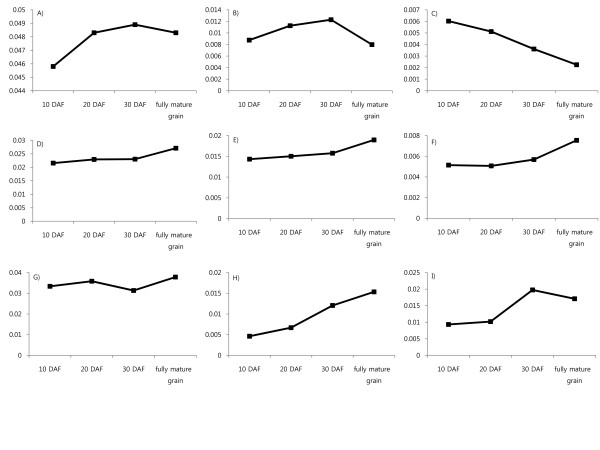
**Composite protein expression profiles of gene function categories**. A) Metabolism (102)* B) Starch biosynthesis (7) C) photosynthesis (11) D) Glycolysis (23) E) TCA-cycle (17) F) Lipid metabolism (19) G) Proteolysis (100) H) late embryogenesis abundant protein (10) I) Chaperone (20) * The total number of non-redundant proteins used to draw the composite profiles is indicated in parentheses.

To characterize global expression trends for proteins involved in specific processes, composite expression profiles were constructed by summing NSpC for each protein in each functional category (according to classification scheme by Bevan et al. (1998) [22]). In addition to 8 functional categories, we constructed a composite expression profile of late-embryogenesis-abundant (LEA) proteins (Figure 4). Proteins involved in metabolic processes increased until 20 DAF, and after that, their levels were maintained in fully mature grains with a slight increase at 30 DAF, suggesting that even after the starch had fully accumulated, proteins involved in metabolic processes continued to be present during the desiccation phase. The expression trends of proteins involved in starch biosynthesis and photosynthesis were in accordance with morphological development. Proteins involved in starch biosynthesis increased until 20 DAF, and then decreased slightly at 30 DAF, followed by rapid decrease for fully mature grains, suggesting that starch accumulation was intensive until 20 DAF, and saturated before 30 DAF. Proteins associated with photosynthesis continuously decreased. For the metabolism, starch biosynthesis, and photosynthesis, the general trends of composite expression profiles during the grain filling stage were similar with the results previous reported by Xu et al. (2008). Due to dynamic proteomic analyses through eight sequential developmental stages until 20 DAF, Xu et al. (2008) could detect fluctuations of protein expression during grain filling stages for other categories, while those detailed expression patterns could not be revealed in this study because of 10 day sampling interval. However, we could reveal their expression patterns in desiccation phase instead. Proteins involved in glycolysis, TCA-cycle, lipid metabolism, and proteolysis increased in fully mature grains. Proteins involved in glycolysis, TCA cycle, and lipid metabolism showed similar expression trends, and the levels of expression for these proteins increased slightly during grain development, being highest for fully mature grains. The roles of glycolysis and the TCA-cycle, which are closely related and provide energy and carbon skeletons for various primary metabolites, increased in fully mature grain. This was also observed for some of the proteins involved in lipid metabolism that have catalytic activity in fatty acids such as acyl-CoA synthetase. The next growth stage of fully mature grain is germination, during which large amounts of energy and nutrition are required, so remobilization of reserves in the endosperm and increases of these proteins in germinating seeds are critical [23]. Thus, the accumulation of such proteins in mature grain may reflect the fact that a certain level of proteins is required for germination. Proteins involved in proteolysis were also increased in fully mature grains, which also may represent preparation for germination. However, the expression trend of proteolysis fluctuated, which may represent the turnover of molecular processes during grain development. The expression trends of LEA proteins increased continuously in fully mature grains, with the majority accumulating during late developmental stages. LEA is associated with desiccation tolerance [24]. Proteins involved in protein folding showed similar trends as LEA proteins, even though their expression levels slightly decreased in fully mature grain. Chaperones in developing grain determine endoplasmic reticulum mediated protein accumulation into protein bodies among other roles [25]. However, the higher levels of expression in the 30 DAF and fully mature grain suggest another role in the desiccation phase. Based on the general role of the chaperone for preventing protein aggregation of misfolding [26], and the similar expression trends of LEA proteins associated with desiccation tolerance, we hypothesized that due to desiccation stress, these chaperones protect the proteins and maintain their expression levels during the desiccation phase in preparation for germination.

## Conclusions

With a label-free shotgun proteomic approach, we were able to identify a rice grain proteome at large scale where physiochemical properties of the identified proteins were unbiased and to monitor protein expression patterns during rice grain development. The comparison analysis of protein expressions, clustering analysis, and GO category enrichment analysis revealed proteome changes in the grain filling stage, desiccation phase, and the fully mature grains. Interestingly, we detected that proteins involved in metabolic process were enriched during the entire developmental stages, suggesting that even in the desiccation phase, changes occurred in molecular levels. Especially, we detected increase of chaperone proteins in the late development stages including desiccation phase and fully mature grains, hypothesizing that the role of chaperone proteins accumulated during the desiccation phase may be associated with conserving proteins associated with germination from desiccation stress. With the advantage that a mass spectrometry based high-throughput proteomic analysis can provide the relative quantities of all of the identified proteins, we were able to draw composite expression profiles which can represent global expression trends for proteins involved in specific processes. Composite expression profiles revealed that proteins required in germination such as glycolysis, TCA-cycle, lipid metabolism, and proteolysis accumulated at higher levels in fully mature grain.

## Materials and methods

### Growth conditions

A Korean commercial variety, Ilpumbyeo (*Japonica *rice), was grown in 15 × 30 cm rows at the Seoul National University Experimental Field. Rice grains were harvested at 10 days after flowering (DAF), 20 DAF, and 30 DAF and then freeze-dried. Air dried fully mature grains which were desiccated during the desiccation phase were harvested at 45 DAF.

### Protein extraction

Proteins were extracted from brown rice powders with extraction buffer (100 mM Tris-HCl pH 8.5, 5 mM DTT, 1 mM EDTA, 2% (m/v) dodecyl-β-maltoside, and 1% (v/v) Plant Proteinase Inhibition Cocktail; Sigma, St. Louis, MO, USA). The suspension was incubated at room temperature for 30 minutes followed by centrifugation at 14,000 g for 15 minutes. The supernatant was retained and filtered through 5 μm membrane filters, and then through 0.45 μm membrane filters (Millipore, Billerica, MA, USA). Extracted proteins were precipitated overnight with 20% (v/v) trichloroacetic acid (TCA), washed three times with cold acetone, and re-solubilized in 8 M Urea/Tris-HCl pH 8.5. Protein concentration was assayed by the 2D-Protein Quant Kit (GE Healthcare, Piscataway, NJ, USA).

### Protein digestion

A total of 500 μg of protein was reduced with Tris(2-carboxyethyl)phosphine hydrochloride (TCEP) by adjusting the protein sample solution to 5 mM TCEP, followed by a 30-minute incubation at room temperature. The reduced sample was carbamidomethylated by adjusting iodoacetamide to 10 mM, followed by a 30-minute incubation at room temperature in the dark. The protein solution was diluted from 8 M urea to 2 M urea with 100 mM Tris-HCl pH 8.5, and then the CaCl_2 _was adjusted to 2 mM. A total of 5 μg of trypsin was added, and the solution was incubated overnight at 37°C. Protein digestion was terminated by adding formic acid to 5%.

### MudPIT analysis

Home-made biphasic columns were prepared with 365 μm o.d. × 100 μm i.d. fused-silica capillaries (Polymicro Technologies, Phoenix, AZ, USA). The tip of each capillary was pulled to 5 μm by a P-2000 laser puller (Sutter Instrument Co., Novato, CA, USA). Each capillary was then packed using a pressure cell under 600 psi of helium with 9 cm of 5 μm reverse phase C18 resin (Phenomenex, Torrance, CA, USA), followed by 4 cm of 5 μm strong cation exchange resin (Phenomenex). Separate desalting columns were prepared with 3 cm of a 365 μm o.d. × 250 μm i.d. fused-silica capillary packed with 5 μm reverse phase C18 resin. Digested peptide samples were loaded onto the desalting column using the same pressure cell that was to be desalted, and then the desalting column was attached to the biphasic column. The sample loaded column was then placed in a home-made ion source, which was connected in-line to a Nanospace SI-2 HPLC (Shiseido, Tokyo, Japan), having a liquid junction with a T-split for the application of electrospray voltage and obtaining the nano scale mobile phase flow rate. Peptides were eluted in a 12-step process by increasing the concentrations of salt solution of 250 mM ammonium formate, followed by an increasing gradient of organic mobile phase at each step, as previously described [27]. The peptide eluent was directly electrosprayed into an LXQ ion trap mass spectrometer (Thermo Fisher Scientific, Waltham, MA, USA). Tandem mass spectra were obtained using Xcalibur 2.0. A parent-ion scan was performed over the range 400-1600 m/z. Automated peak recognition, dynamic exclusion, and MS/MS-ion scanning of the top five most intense parent ions were performed.

### Identification of the rice grain proteome

Each peptide from the MS/MS spectra was searched by MASCOT against the TIGR Rice Pseudomolecule protein database Release V6.1 (http://rice.plantbiology.msu.edu/annotation_pseudo_current.shtml), as well as general contaminant lists. The search parameters were as follows: tryptic digests with one possible missed cleavage, carbamidomethylation set as a fixed amino acid modification, oxidation considered a variable amino acid modification, averaged mass values, peptide mass tolerance of +/- 1.4 Da, and fragment mass tolerance of +/- 0.8 Da. The MASCOT generic file was used, and the analytical instrument was ESI-TRAP. MASCOT output was processed using PANORAMICS, a probability-based program that determines the false-positive rate of identification [28].

### Comparative analysis of relative protein abundances

The output of PANORAMICS was exported to Microsoft Excel to calculate normalized spectral counts (NSpC)[14,16]. Th NSpC for each protein *k *is given by

$${\left(\text{NSpC}\right)}_{\text{k}}=\frac{{\left(\frac{\text{SpC}}{\text{L}}\right)}_{\text{k}}}{\sum_{\text{i}=1}^{\text{n}}{\left(\frac{\text{SpC}}{\text{L}}\right)}_{\text{i}}}$$

where the total number of MS/MS spectra matching peptides from protein *k *(SpC) is divided by the protein's length (*L*), then divided by SpC/*L *for all N proteins in the experiment.

### Bioinformatics analysis

GO annotations of the rice proteins were retrieved the from TIGR Rice Pseudomolecule protein database Release V6.1. The GO enrichment analysis was performed in agriGO (http://bioinfo.cau.edu.cn/agriGO/)[19] with default parameters using the rice whole genome as the background/reference.

The hierarchical clustering analysis were conducted with the Cluster 3.0 software using centered correlation and the average linkage procedure and the tree was visualized with the Java Treeview 1.1.1[29]. Composite expression profile analysis was performed by summing averages of NSpC for all proteins of a given functional category at each of the four developmental stages [30].

## Competing interests

The authors declare that they have no competing interests.

## Authors' contributions

JL performed the experiments and drafted the manuscript.

HJK contributed to data analysis and interpretation and manuscript editing

JL and HJK read and approved the final manuscript.

## Supplementary Material

Additional file 1 **List of 4,172 proteins identified through all MudPIT experiments by merging the identified protein lists from all three biological replicates for four time points**. Click here for file

Additional file 2 **Relative abundance (NSpC) of 484 differentially expressed proteins during rice grain development**. Click here for file

Additional file 3 **Clustering 484 differentially expressed proteins and their GO annotations**. Click here for file

Additional file 4 **Table S1**. Enriched GO terms of molecular functions and cellular components in the constructed rice grain proteome Click here for file

Additional file 5 **Table S2**. Enriched GO terms of biological processes in the clusters. Click here for file
