# Supplementary material for: A label-free quantitative shotgun proteomics analysis of rice grain development
Source: Proteome Sci. 2011 Sep 30;9:61. doi: 10.1186/1477-5956-9-61 (PMC3190340; doi:10.1186/1477-5956-9-61)
Supplement: Additional file 4 — Table S1. Enriched GO terms of molecular functions and cellular components in the constructed rice grain proteome [file 1477-5956-9-61-S4.DOC]

Table S1. Enriched GO terms of molecular functions and cellular components in the constructed rice grain proteome

| GO term | Ontology^a)^ | Description | Number in the identified rice grain proteome | Number in rice genome | Adjusted *p*-value^b)*^ |
| --- | --- | --- | --- | --- | --- |
| GO:0005198 | F | structural molecule activity | 163 | 531 | 3.30E-26 |
| GO:0016462 | F | pyrophosphatase activity | 185 | 747 | 3.00E-18 |
| GO:0016817 | F | hydrolase activity, acting on acid anhydrides | 191 | 787 | 4.50E-18 |
| GO:0016818 | F | hydrolase activity, acting on acid anhydrides, in phosphorus-containing anhydrides | 186 | 760 | 4.50E-18 |
| GO:0017111 | F | nucleoside-triphosphatase activity | 170 | 714 | 1.70E-15 |
| GO:0008135 | F | translation factor activity, nucleic acid binding | 33 | 77 | 4.60E-10 |
| GO:0000166 | F | nucleotide binding | 613 | 3794 | 1.50E-09 |
| GO:0030234 | F | enzyme regulator activity | 59 | 263 | 5.40E-05 |
| GO:0008289 | F | lipid binding | 25 | 88 | 0.00052 |
| GO:0003824 | F | catalytic activity | 1829 | 13462 | 0.0025 |
| GO:0005215 | F | transporter activity | 169 | 1110 | 0.029 |
| GO:0005737 | C | cytoplasm | 465 | 1271 | 4.70E-99 |
| GO:0044444 | C | cytoplasmic part | 322 | 960 | 3.40E-60 |
| GO:0032991 | C | macromolecular complex | 390 | 1365 | 3.10E-52 |
| GO:0030529 | C | ribonucleoprotein complex | 153 | 503 | 1.50E-24 |
| GO:0005840 | C | ribosome | 134 | 456 | 3.40E-20 |
| GO:0031967 | C | organelle envelope | 57 | 137 | 3.50E-16 |
| GO:0005622 | C | intracellular | 754 | 4460 | 8.00E-15 |
| GO:0031975 | C | envelope | 58 | 158 | 1.20E-13 |
| GO:0044424 | C | intracellular part | 646 | 3831 | 5.80E-13 |
| GO:0012505 | C | endomembrane system | 44 | 110 | 4.90E-12 |
| GO:0005794 | C | Golgi apparatus | 29 | 56 | 1.70E-11 |
| GO:0005739 | C | mitochondrion | 48 | 131 | 1.70E-11 |
| GO:0005829 | C | cytosol | 19 | 33 | 1.10E-08 |
| GO:0044464 | C | cell part | 966 | 6353 | 1.40E-08 |
| GO:0005623 | C | cell | 966 | 6353 | 1.40E-08 |
| GO:0016020 | C | membrane | 357 | 2185 | 1.10E-06 |
| GO:0005635 | C | nuclear envelope | 14 | 27 | 8.20E-06 |
| GO:0005773 | C | vacuole | 7 | 9 | 0.00012 |
| GO:0005783 | C | endoplasmic reticulum | 25 | 91 | 0.00075 |
| GO:0005777 | C | peroxisome | 7 | 15 | 0.0093 |
| GO:0042579 | C | microbody | 7 | 15 | 0.0093 |
| GO:0005886 | C | plasma membrane | 12 | 37 | 0.0093 |
| GO:0009579 | C | thylakoid | 18 | 72 | 0.019 |
| GO:0044428 | C | nuclear part | 23 | 103 | 0.024 |

1. F: molecular function, C: cellular components
2. Based on the Fisher’s statistical method and the Yekutieli FDR multiple test correction method
