# Supplementary material for: A label-free quantitative shotgun proteomics analysis of rice grain development
Source: Proteome Sci. 2011 Sep 30;9:61. doi: 10.1186/1477-5956-9-61 (PMC3190340; doi:10.1186/1477-5956-9-61)
Supplement: Additional file 5 — Table S2. Enriched GO terms of biological processes in the clusters. [file 1477-5956-9-61-S5.DOC]

Table S2. Enriched GO terms of biological processes in the groups.

| GO term | Group | Description | Number in the identified rice grain proteome | Number in rice genome | Adjusted *p*-value^a)^ |
| --- | --- | --- | --- | --- | --- |
| GO:0008152 | group 1 | metabolic process | 79 | 9541 | 1E-08 |
| GO:0019684 | group 1 | photosynthesis, light reaction | 7 | 49 | 2.9E-08 |
| GO:0015979 | group 1 | photosynthesis | 9 | 125 | 3E-08 |
| GO:0009765 | group 1 | photosynthesis, light harvesting | 5 | 18 | 1.5E-07 |
| GO:0009987 | group 1 | cellular process | 63 | 7980 | 3E-07 |
| GO:0044237 | group 1 | cellular metabolic process | 55 | 6576 | 3E-07 |
| GO:0006412 | group 1 | translation | 14 | 681 | 0.000003 |
| GO:0006091 | group 1 | generation of precursor metabolites and energy | 9 | 246 | 0.000004 |
| GO:0044238 | group 1 | primary metabolic process | 55 | 7728 | 0.000021 |
| GO:0044281 | group 1 | small molecule metabolic process | 13 | 884 | 0.00021 |
| GO:0006520 | group 1 | cellular amino acid metabolic process | 8 | 319 | 0.00022 |
| GO:0034641 | group 1 | cellular nitrogen compound metabolic process | 9 | 461 | 0.0004 |
| GO:0044106 | group 1 | cellular amine metabolic process | 8 | 352 | 0.0004 |
| GO:0019538 | group 1 | protein metabolic process | 31 | 4043 | 0.00044 |
| GO:0044267 | group 1 | cellular protein metabolic process | 25 | 2971 | 0.00045 |
| GO:0006519 | group 1 | cellular amino acid and derivative metabolic process | 8 | 372 | 0.00045 |
| GO:0009308 | group 1 | amine metabolic process | 8 | 399 | 0.00069 |
| GO:0019752 | group 1 | carboxylic acid metabolic process | 8 | 433 | 0.001 |
| GO:0043436 | group 1 | oxoacid metabolic process | 8 | 433 | 0.001 |
| GO:0042180 | group 1 | cellular ketone metabolic process | 8 | 437 | 0.001 |
| GO:0006082 | group 1 | organic acid metabolic process | 8 | 434 | 0.001 |
| GO:0009058 | group 1 | biosynthetic process | 24 | 3274 | 0.0032 |
| GO:0009056 | group 1 | catabolic process | 7 | 415 | 0.0041 |
| GO:0044248 | group 1 | cellular catabolic process | 6 | 309 | 0.0047 |
| GO:0009057 | group 1 | macromolecule catabolic process | 6 | 316 | 0.0049 |
| GO:0043170 | group 1 | macromolecule metabolic process | 38 | 6374 | 0.0049 |
| GO:0044260 | group 1 | cellular macromolecule metabolic process | 33 | 5328 | 0.0052 |
| GO:0044265 | group 1 | cellular macromolecule catabolic process | 5 | 251 | 0.01 |
| GO:0044249 | group 1 | cellular biosynthetic process | 21 | 3054 | 0.011 |
| GO:0006118 | group 1 | electron transport | 10 | 1055 | 0.018 |
| GO:0010467 | group 1 | gene expression | 17 | 2459 | 0.024 |
| GO:0016064 | group 2 | immunoglobulin mediated immune response | 5 | 5 | 1.1E-11 |
| GO:0002437 | group 2 | inflammatory response to antigenic stimulus | 5 | 5 | 1.1E-11 |
| GO:0016068 | group 2 | type 1 hypersensitivity | 5 | 5 | 1.1E-11 |
| GO:0002443 | group 2 | leukocyte mediated immunity | 5 | 5 | 1.1E-11 |
| GO:0019724 | group 2 | B cell mediated immunity | 5 | 5 | 1.1E-11 |
| GO:0002438 | group 2 | acute inflammatory response to antigenic stimulus | 5 | 5 | 1.1E-11 |
| GO:0006954 | group 2 | inflammatory response | 5 | 5 | 1.1E-11 |
| GO:0002526 | group 2 | acute inflammatory response | 5 | 5 | 1.1E-11 |
| GO:0002460 | group 2 | adaptive immune response based on somatic recombination of immune receptors built from immunoglobulin superfamily domains | 5 | 5 | 1.1E-11 |
| GO:0002449 | group 2 | lymphocyte mediated immunity | 5 | 5 | 1.1E-11 |
| GO:0002250 | group 2 | adaptive immune response | 5 | 5 | 1.1E-11 |
| GO:0002524 | group 2 | hypersensitivity | 5 | 5 | 1.1E-11 |
| GO:0002252 | group 2 | immune effector process | 5 | 5 | 1.1E-11 |
| GO:0006955 | group 2 | immune response | 5 | 12 | 7.9E-09 |
| GO:0002376 | group 2 | immune system process | 5 | 15 | 2.8E-08 |
| GO:0009611 | group 2 | response to wounding | 5 | 21 | 1.7E-07 |
| GO:0009605 | group 2 | response to external stimulus | 5 | 31 | 1.3E-06 |
| GO:0009308 | group 2 | amine metabolic process | 10 | 399 | 0.000034 |
| GO:0006082 | group 2 | organic acid metabolic process | 10 | 434 | 0.000061 |
| GO:0019752 | group 2 | carboxylic acid metabolic process | 10 | 433 | 0.000061 |
| GO:0043436 | group 2 | oxoacid metabolic process | 10 | 433 | 0.000061 |
| GO:0042180 | group 2 | cellular ketone metabolic process | 10 | 437 | 0.000062 |
| GO:0033036 | group 2 | macromolecule localization | 8 | 406 | 0.0014 |
| GO:0006725 | group 2 | cellular aromatic compound metabolic process | 5 | 139 | 0.0016 |
| GO:0008152 | group 2 | metabolic process | 59 | 9541 | 0.0017 |
| GO:0006520 | group 2 | cellular amino acid metabolic process | 7 | 319 | 0.0017 |
| GO:0044106 | group 2 | cellular amine metabolic process | 7 | 352 | 0.0029 |
| GO:0044281 | group 2 | small molecule metabolic process | 11 | 884 | 0.0038 |
| GO:0006519 | group 2 | cellular amino acid and derivative metabolic process | 7 | 372 | 0.0038 |
| GO:0006457 | group 2 | protein folding | 5 | 198 | 0.0065 |
| GO:0034641 | group 2 | cellular nitrogen compound metabolic process | 7 | 461 | 0.012 |
| GO:0006810 | group 2 | transport | 14 | 1582 | 0.016 |
| GO:0051234 | group 2 | establishment of localization | 14 | 1582 | 0.016 |
| GO:0046483 | group 2 | heterocycle metabolic process | 5 | 248 | 0.016 |
| GO:0051179 | group 2 | localization | 14 | 1601 | 0.017 |
| GO:0045184 | group 2 | establishment of protein localization | 5 | 287 | 0.027 |
| GO:0015031 | group 2 | protein transport | 5 | 287 | 0.027 |
| GO:0005975 | group 2 | carbohydrate metabolic process | 9 | 859 | 0.028 |
| GO:0008104 | group 2 | protein localization | 5 | 298 | 0.029 |
| GO:0051649 | group 2 | establishment of localization in cell | 5 | 307 | 0.032 |
| GO:0006952 | group 2 | defense response | 6 | 435 | 0.032 |
| GO:0009987 | group 2 | cellular process | 44 | 7980 | 0.032 |
| GO:0051641 | group 2 | cellular localization | 5 | 315 | 0.034 |
| GO:0044281 | group 3 | small molecule metabolic process | 36 | 884 | 1.7E-20 |
| GO:0019752 | group 3 | carboxylic acid metabolic process | 18 | 433 | 7.5E-11 |
| GO:0008152 | group 3 | metabolic process | 99 | 9541 | 7.5E-11 |
| GO:0043436 | group 3 | oxoacid metabolic process | 18 | 433 | 7.5E-11 |
| GO:0042180 | group 3 | cellular ketone metabolic process | 18 | 437 | 7.5E-11 |
| GO:0006082 | group 3 | organic acid metabolic process | 18 | 434 | 7.5E-11 |
| GO:0044238 | group 3 | primary metabolic process | 81 | 7728 | 7.2E-10 |
| GO:0005975 | group 3 | carbohydrate metabolic process | 22 | 859 | 2.3E-09 |
| GO:0044237 | group 3 | cellular metabolic process | 66 | 6576 | 9.7E-08 |
| GO:0009987 | group 3 | cellular process | 75 | 7980 | 1.5E-07 |
| GO:0044262 | group 3 | cellular carbohydrate metabolic process | 13 | 369 | 3E-07 |
| GO:0006006 | group 3 | glucose metabolic process | 8 | 110 | 7.7E-07 |
| GO:0009150 | group 3 | purine ribonucleotide metabolic process | 7 | 77 | 1.1E-06 |
| GO:0009152 | group 3 | purine ribonucleotide biosynthetic process | 7 | 77 | 1.1E-06 |
| GO:0009259 | group 3 | ribonucleotide metabolic process | 7 | 80 | 1.2E-06 |
| GO:0009260 | group 3 | ribonucleotide biosynthetic process | 7 | 80 | 1.2E-06 |
| GO:0055086 | group 3 | nucleobase, nucleoside and nucleotide metabolic process | 9 | 171 | 1.2E-06 |
| GO:0006163 | group 3 | purine nucleotide metabolic process | 7 | 83 | 1.3E-06 |
| GO:0006164 | group 3 | purine nucleotide biosynthetic process | 7 | 83 | 1.3E-06 |
| GO:0019318 | group 3 | hexose metabolic process | 8 | 127 | 1.4E-06 |
| GO:0006519 | group 3 | cellular amino acid and derivative metabolic process | 12 | 372 | 1.5E-06 |
| GO:0009206 | group 3 | purine ribonucleoside triphosphate biosynthetic process | 6 | 60 | 2.6E-06 |
| GO:0009144 | group 3 | purine nucleoside triphosphate metabolic process | 6 | 60 | 2.6E-06 |
| GO:0009145 | group 3 | purine nucleoside triphosphate biosynthetic process | 6 | 60 | 2.6E-06 |
| GO:0009201 | group 3 | ribonucleoside triphosphate biosynthetic process | 6 | 60 | 2.6E-06 |
| GO:0009199 | group 3 | ribonucleoside triphosphate metabolic process | 6 | 60 | 2.6E-06 |
| GO:0009205 | group 3 | purine ribonucleoside triphosphate metabolic process | 6 | 60 | 2.6E-06 |
| GO:0009117 | group 3 | nucleotide metabolic process | 8 | 144 | 2.6E-06 |
| GO:0005996 | group 3 | monosaccharide metabolic process | 8 | 143 | 2.6E-06 |
| GO:0006753 | group 3 | nucleoside phosphate metabolic process | 8 | 144 | 2.6E-06 |
| GO:0009141 | group 3 | nucleoside triphosphate metabolic process | 6 | 62 | 0.000003 |
| GO:0009142 | group 3 | nucleoside triphosphate biosynthetic process | 6 | 62 | 0.000003 |
| GO:0009165 | group 3 | nucleotide biosynthetic process | 7 | 108 | 4.6E-06 |
| GO:0009058 | group 3 | biosynthetic process | 36 | 3274 | 6.2E-06 |
| GO:0044249 | group 3 | cellular biosynthetic process | 34 | 3054 | 8.5E-06 |
| GO:0034641 | group 3 | cellular nitrogen compound metabolic process | 12 | 461 | 8.5E-06 |
| GO:0006091 | group 3 | generation of precursor metabolites and energy | 9 | 246 | 0.000012 |
| GO:0006520 | group 3 | cellular amino acid metabolic process | 10 | 319 | 0.000012 |
| GO:0046483 | group 3 | heterocycle metabolic process | 9 | 248 | 0.000012 |
| GO:0006066 | group 3 | alcohol metabolic process | 8 | 189 | 0.000015 |
| GO:0009056 | group 3 | catabolic process | 11 | 415 | 0.000017 |
| GO:0044106 | group 3 | cellular amine metabolic process | 10 | 352 | 0.000027 |
| GO:0019320 | group 3 | hexose catabolic process | 6 | 98 | 0.000032 |
| GO:0006007 | group 3 | glucose catabolic process | 6 | 98 | 0.000032 |
| GO:0046365 | group 3 | monosaccharide catabolic process | 6 | 98 | 0.000032 |
| GO:0046164 | group 3 | alcohol catabolic process | 6 | 103 | 0.000041 |
| GO:0044275 | group 3 | cellular carbohydrate catabolic process | 6 | 103 | 0.000041 |
| GO:0044248 | group 3 | cellular catabolic process | 9 | 309 | 0.000059 |
| GO:0006412 | group 3 | translation | 13 | 681 | 0.000064 |
| GO:0009308 | group 3 | amine metabolic process | 10 | 399 | 0.000066 |
| GO:0009057 | group 3 | macromolecule catabolic process | 9 | 316 | 0.000066 |
| GO:0044283 | group 3 | small molecule biosynthetic process | 8 | 250 | 0.000088 |
| GO:0044265 | group 3 | cellular macromolecule catabolic process | 8 | 251 | 0.000088 |
| GO:0044282 | group 3 | small molecule catabolic process | 6 | 127 | 0.00012 |
| GO:0032787 | group 3 | monocarboxylic acid metabolic process | 5 | 76 | 0.00012 |
| GO:0016052 | group 3 | carbohydrate catabolic process | 6 | 139 | 0.00019 |
| GO:0006732 | group 3 | coenzyme metabolic process | 5 | 96 | 0.00036 |
| GO:0016053 | group 3 | organic acid biosynthetic process | 6 | 159 | 0.00038 |
| GO:0046394 | group 3 | carboxylic acid biosynthetic process | 6 | 159 | 0.00038 |
| GO:0019538 | group 3 | protein metabolic process | 33 | 4043 | 0.0022 |
| GO:0051186 | group 3 | cofactor metabolic process | 5 | 143 | 0.0022 |
| GO:0044085 | group 3 | cellular component biogenesis | 6 | 288 | 0.0086 |
| GO:0006457 | group 3 | protein folding | 5 | 198 | 0.0092 |
| GO:0043170 | group 3 | macromolecule metabolic process | 43 | 6374 | 0.014 |
| GO:0044271 | group 3 | cellular nitrogen compound biosynthetic process | 5 | 225 | 0.016 |
| GO:0006508 | group 3 | proteolysis | 12 | 1187 | 0.027 |
| GO:0016043 | group 3 | cellular component organization | 6 | 377 | 0.03 |
| GO:0006807 | group 3 | nitrogen compound metabolic process | 22 | 2897 | 0.034 |

1. Based on the Fisher’s statistical method and the Yekutieli FDR multiple test correction method
